# Cranio-cervical and traumatic brain injury patterns—do they differ between electric bicycle, bicycle, and motorcycle-induced accidents?

**DOI:** 10.1007/s00068-024-02510-1

**Published:** 2024-04-09

**Authors:** Thomas Rauer, Felix Karl-Ludwig Klingebiel, Adrian Lühring, Alexander Küffer, Anna-Sophie Hofer, Raphael Marco Ferrari, Michael Kupka, Hans-Christoph Pape

**Affiliations:** 1 Department of Trauma Surgery, University Hospital Zurich, 8091 Zurich, Switzerland; 2 Department of Surgical Research, Harald Tscherne Laboratory for Orthopaedic and Trauma Research, Zurich University Hospital, Zurich, Switzerland; 3 Medical University of Innsbruck, 6020 Innsbruck, Austria; 4 Department of Neurosurgery, University Hospital Zurich, 8091 Zurich, Switzerland; 5 Department of Neurosurgery, Medical University Innsbruck, 6020 Innsbruck, Austria; 6 Department of Cranio-Maxillo-Facial and Oral Surgery, University Hospital Zurich, 8091 Zurich, Switzerland; 7 Department of Neuroradiology, University Hospital Zurich, 8091 Zurich, Switzerland

**Keywords:** E-bike injuries, Traumatic brain injury, Outcome, Helmet protection

## Abstract

**Purpose:**

With the growing technical options of power transmission and energy-saving options in electric drives, the number of E-bike-related accidents especially in an elderly population has increased. The aim of the current study was to compare if the increased velocity in comparison to conventional bikes translates into different injury patterns in the cranio-cervical and head region.

**Methods:**

A retrospective cohort study was performed in patients admitted to our level one trauma center between 2009 and 2019 after being involved in an accident with either an E-bike, bicycle, or motorcycle and suffered cranio-cervical or traumatic brain injury. Outcomes: cranio-cervical/intracranial injury pattern. Data interpretation was conducted in an interdisciplinary approach.

**Results:**

From 3292 patients treated in this period, we included 1068 patients. E-bikers were significantly older than bicyclists (or motorcyclists) and lay between the other two groups in terms of helmet use. Overall injury patterns of E-bikers resembled those found in motorcyclists rather than in bicyclists. E-bikers had a higher incidence of different cerebral bleedings, especially if no helmet was worn. Helmet protection of E-bikers resulted in a comparable frequency of intracranial bleeding to the helmeted bicyclists.

**Conclusion:**

The overall pattern of head and cervical injuries in E-bikers resembles more to that of motorcyclists than that of bicyclists. As they are used by a more senior population, multiple risk factors apply in terms of complications and secondary intracranial bleeding. Our study suggests that preventive measures should be reinforced, i.e., use of helmets to prevent from intracranial injury.

**Supplementary Information:**

The online version contains supplementary material available at 10.1007/s00068-024-02510-1.

## Purpose

Electric bicycles (E-bikes) are designed for riding at a higher speed than conventional bicycles, with a velocity comparable to that of small motorcycles in dependence on the motor assistance provided [1, 2]. This motor support of E-bikes is particularly attractive for older riders, as it allows them to regain a higher degree of mobility.

The popularity of E-bikes as a means of transportation has increased significantly in recent years, which has subsequently led to a substantial increase in E-bike-related traffic accidents [3–5]. Therefore, the topic of accident prevention and traffic safety has also gained a high level of significance.

While some studies have addressed accident characteristics [6] or riding behavior [7], others have examined injury severity [3, 5, 8–10] or injury patterns associated with E-bike accidents [2, 4, 5, 8–12]. However, to date, only few studies have compared E-bike-related injuries with injury patterns of conventional bicyclists or motorcyclists [2, 3, 11]. A recent study comparing the injury patterns of E-bikers, bicyclists, and motorcyclists showed that the overall injury pattern between E-bikers and conventional bicyclists is comparable and suggested that the differences in injury patterns compared to motorcycle accidents might be related to differences in speed at the time of the accident, different protective gear, and vehicle architecture [2]. Interestingly, a higher rate of craniocerebral trauma was found in E-bikers involved in accidents compared to bicyclists [2].

The aim of the present study was to analyze the injury patterns and severity of cranio-cervical and traumatic brain injuries suffered in E-bike accidents compared to conventional bicycle and motorcycle accidents to further fill the gap in the available literature on this current topic with high relevance for everyday life. We hypothesize that the cranio-cervical and traumatic brain injuries of E-bikers are more similar to the corresponding injury patterns of motorcycle accidents than those sustained by conventional bicyclists, mainly due to a higher accident speed.

## Methods

The present study is a monocentric retrospective cohort study, which was approved by the local Institutional Review Board (PB_2016-01888). It follows the STROBE (Strengthening the Reporting of Observational Studies in Epidemiology) guidelines for reporting observational studies [13].

### Inclusion and exclusion criteria

All patients included in this study were treated at the University Hospital of Zurich, a level I academic trauma center, after involvement in a traffic accident between 2009 and 2018. All medical data were retrieved from electronic medical records routinely collected during hospitalization and analyzed retrospectively. Patients were followed up until hospital discharge.

Patients were included in this study if they were riders of an E-bike, conventional bicycle, or motorcycle who sustained and were treated for isolated or non-isolated cranio-cervical and/or traumatic brain injuries sustained in a traffic accident. Only patients with 16 years of age or older were included. Patients who were hit by a bicycle, pillion riders, and patients with injuries resulting from an accident with a motorized stand-up scooter were excluded. Patients who had an accident abroad were also excluded unless they had been transferred to our trauma center from a neighboring country within 24 h of the accident. We further excluded patients in case of more than 10% missing values, patients who had undergone elective surgery after a bicycle accident without first presenting to our hospital for initial treatment, and outpatients due to a lack of detailed information on accident mechanisms and medical clarification.

All patients were stratified based on the vehicle ridden during the injury: E-bike group (E), bicycle group (B), or motorcycle group (M).

The E-bike group included both E-bikes with motor assistance up to 25 km/h and those with assistance up to 45 km/h, as it was not possible to retrospectively distinguish between these two types of assistance based on the available data set. The bicycle group subsumed conventional bicycles and mountain bikes. In the motorcycle group, mopeds were included in addition to classic motorcycles.

### Collection of data

Patients were identified in the hospital’s computerized patient database using the corresponding International Classification of Diseases (ICD) code for traffic accidents (ICD V99). Only E-bike, conventional bicycle, and motorcycle accidents were selected from this pooled data. The medical database allowed immediate retrieval of previous diagnostic reports, clinical findings, treatments received, and other relevant data that could be analyzed. Because not all possible rider accidents were marked by the correct ICD code and not every report contained accurate information about the vehicle or accident type, patients with incomplete documentation were called and interviewed. Further details of patient recruitment and measures against biases were previously published [2].

Data collected included sex, age, BMI, helmet use, time of accident, injury distribution, and severity of cranio-cervical and traumatic brain injuries. Injuries were divided by anatomic region into the neurocranium (including traumatic brain injuries), face (viscerocranium), and cervical spine. Traumatic brain injuries (TBI) were further divided based on the initial Glasgow Coma Scale score into mild (initial GCS score 13–15), moderate (initial GCS score 9–12), and severe (initial GCS score 3–8). For subgroup analysis, patients were subdivided into three age groups: young (16–30 years old), adult (31–59 years old), elderly (60 years and older). Furthermore, seasonal influences were considered by subgroup analysis for accidents during spring (March–May), summer (June–August), fall (September–November), and winter (December–February).

### Statistical analysis

The primary analysis of the data is based on descriptive statistics to present comparative measurements between the three groups and a subgroup analysis considering helmet usage and elderly state. The bicycle and motorcycle group were not compared to each other since this does not correspond to the focus of our study. Continuous variables are presented as means ± standard deviation (SD), and categorical variables are presented as numbers and percentages (%). Statistical analysis was performed using R (R Core Team [2019], R Foundation for Statistical Computing, Vienna, Austria [https://www.R-project.org)]). Figures were created using the ggplot2 package in R. Normal distribution of data was confirmed by histogram visualization. A chi-squared test was used for categorical values, and a one-way ANOVA was performed for continuous variables. *p*-values were adjusted for multiple testing, using the false discovery rate (FDR). Adjusted odds ratios were calculated by the use of linear regression analysis. An automated matched pair analysis (4:1 ratio to ensure enhance power) was performed in between the bike and E-bike groups to validate previous results despite baseline differences using the “MatchIt” package in R. Propensity score matching was performed for age, sex, and helmet use. Results were considered statistically significant if the adjusted *p*-value was < 0.05.

The results of the study were interpreted and discussed by an interdisciplinary research group consisting of experienced specialists in the disciplines of Trauma Surgery, Neurosurgery, Cranio-Maxillo-Facial and Oral Surgery, and Neuroradiology. This emphasizes the clinical relevance of the reported results and conclusions.

## Results

### Demographics

**Fig. 1:**
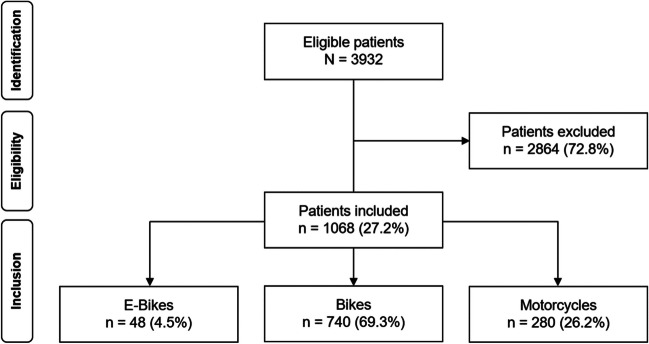
Inclusion flowchart

Out of 3932 eligible patients, 1068 met the inclusion criteria. Forty-eight patients (4.5%) had E-bike (E)-related injuries, 740 (69.3%) had bicycle (B)-related injuries, and 280 (26.2%) had motorcycle (M)-related injuries (Fig. 1).

**Fig. 2:**
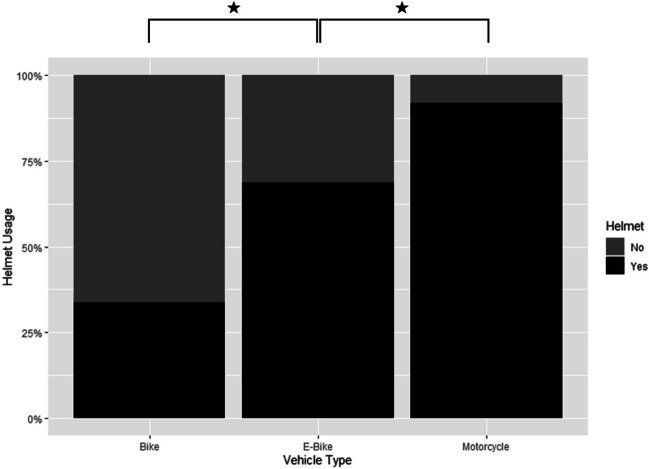
Helmet usage

The E-bike group was significantly older at the time of accident with a mean age of 54.96 (± 16.34) years compared to the bicycle group (42.5 ± 16.69 years) and the motorcycle group (40.19 ± 16.10 years; E vs. B: *p* < 0.006; E vs. M: *p* < 0.006). The distribution of age groups (young, adult, elderly) differed significantly between patients using E-bikes and bicycles (*p* < 0.006) as well as E-bikes and motorcycles (*p* < 0.006). Elderly people (≥ 60 years) were mostly represented in the E-bike group with 41.7%, less in the bicycle (17.4%) and least in the motorcycle group (11.8%). On the other hand, we observed an overall tendency for young riders to use bicycles (29.6%) and motorcycles (31.4%), with only three young patients using an E-bike (6.2%). Adults were distributed equally among the three groups (E, 52.1%; B, 53.0%; M, 56.8%). The sex distribution showed a predominance of male patients in the motorcycle group (89.3%), whereas sex distribution was more balanced in the bicyclist (male, 69.1%) and E-biker group (male, 64.6%, E vs. M: *p* < 0.006; E vs. B: *p* = 0.946). The body mass index (BMI) was highest in patients using the motorcycle (BMI 25.29), followed by E-bikers (BMI 24.83) and bicyclists (BMI 23.65, E vs. B: *p* = 0.0807; E vs. M: *p* = 0.816). Helmet usage differed significantly (E vs. B and E vs. M: *p* < 0.006) among the three vehicle groups with motorcyclists using a helmet in 91.8%, E-bikers in 68.8%, and bicyclists only in 33.8% of cases (Table 1, Fig. 2). Further injury circumstances are presented in the supplements (S1).

**Table 1:** Demographics

	Bike (B)	E-bike (E)	Motorcycle (M)	*p*-value (adj.) (E vs. B)	*p*-value (adj.) (E vs. M)
*N*	740	48	280		
Sex (male) (*n*, %)	511 (69.1%)	31 (64.6%)	250 (89.3%)	0.946	** < 0.006**
Age (mean/SD)	42.50 (± 16.69)	54.96 (± 16.34)	40.19 (± 16.10)	** < 0.006**	** < 0.006**
BMI (mean/SD)	23.65 (± 3.24)	24.83 (± 3.95)	25.29 (± 3.72)	0.0807	0.816
Helmet (yes) (*n*, %)	248 (33.8%)	33 (68.8%)	257 (91.8%)	** < 0.006**	** < 0.006**

### Overview of traumatic craniocerebral injuries

The mean severity of traumatic brain injuries (TBI) according to the Glasgow Coma Scale (GCS) did not differ significantly between groups (E vs. B: *p* = 0.419; E vs. M: *p* = 0.849). Most patients suffered a mild TBI (E, 77.1%; B, 85.7%; M, 73.2%), followed by moderate (E, 14.6%; B, 6.1; M, 10.0%) and severe TBI (E, 4.2%; B, 4.6%; M, 11.1%). However, the median GCS at admission was lower in patients with an E-bike-related injury compared to bicycle injuries without statistical significance after adjustment for multiple testing (GCS: E = 13.27 ± 2.88; B = 14.05 ± 2.27), and was comparable to the motorcycle group (M, 13.16 ± 3.41).

**Fig. 3:**
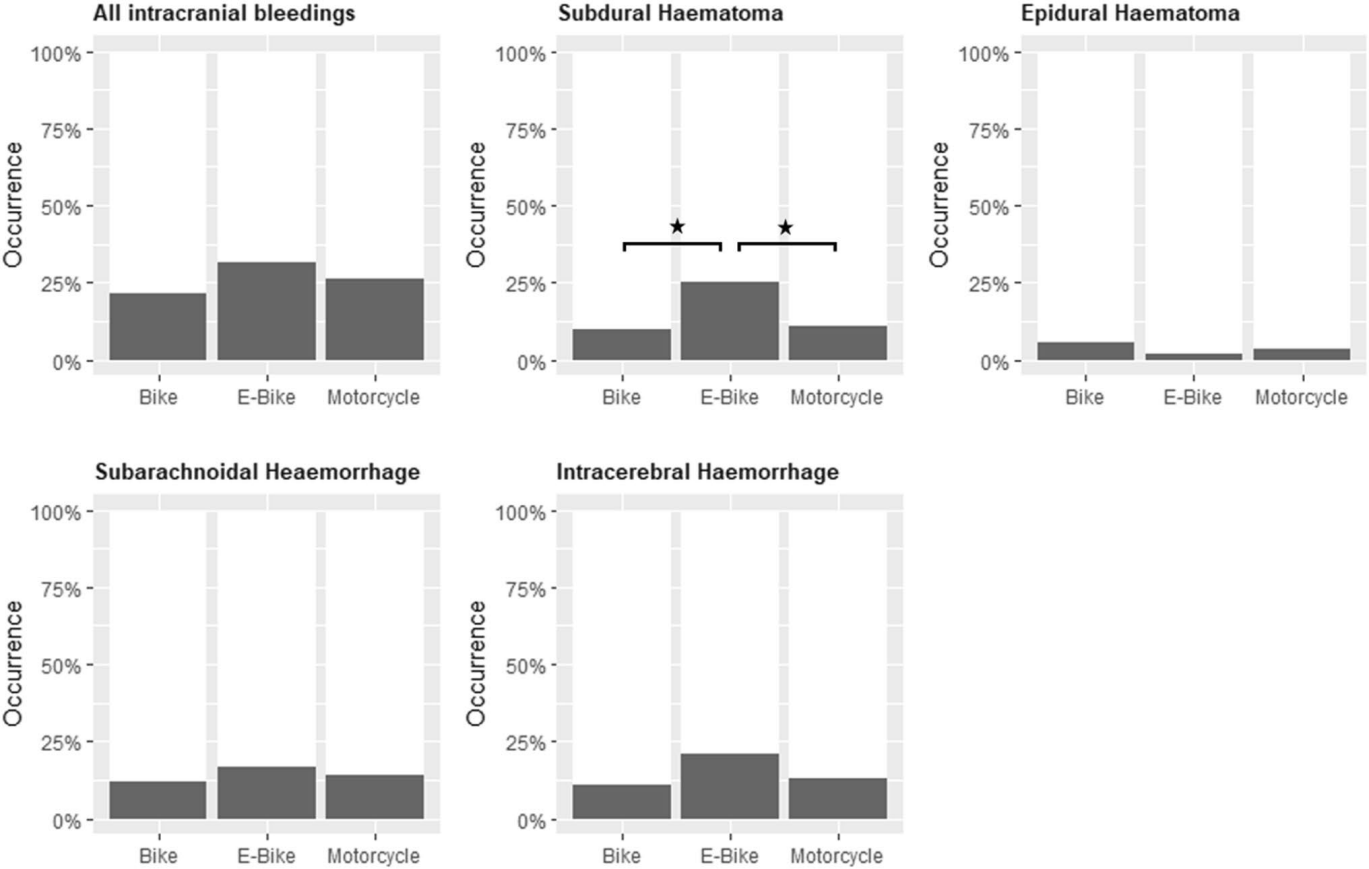
Occurrence of intracranial bleedings for each vehicle type

Overall, intracerebral bleeding occurrence (subdural hematoma [SDH], epidural hematoma [EDH], subarachnoid hemorrhage [SAH], and intracerebral hemorrhage [ICH]) did not differ significantly between the three groups (E, 31.2%; B, 21.2%; M, 26.1%); however, the presence of an SDH was significantly higher (E vs. B: *p* = 0.0107; E vs. M: *p* = 0.0495) after suffering an E-bike-related traumatic brain injury, with a concomitant significantly higher mean AIS of the SDH. Other intracerebral or cranial injuries such as EDH, SAH, ICH, cerebral edema, diffuse axonal injury, vascular injuries (intracerebral venous sinus injuries, arterial injuries), presence of pneumocephalus, and osseous injuries did not differ between the three groups after adjustment for multiple testing (*p* > 0.05) (S2, Fig. 3).

### Influence of helmet usage on craniocerebral injury pattern and severity

A subgroup analysis of the influence of helmet usage on the craniocerebral injury pattern and severity was performed for each vehicle class and compared between groups. TBI severity was significantly higher in the E-bike group (*p* = 0.0007), and the mean GCS was significantly lower (GCS: mean 11.27 vs. 14.18, *p* = 0.006) in the non-helmet (NH) E-bike subgroup compared to the helmet (H) E-bike subgroup or compared to bicyclists without head protection (TBI: *p* = 0.006; GCS, 14.0, *p* < 0.006) at the time of accident (S3). The GCS at admission and severity of TBI of E-bikers wearing a helmet was comparable to cyclists wearing a helmet (TBI: *p* = 1.0; GCS, 14.18 vs. 13.19, *p* = 1.0).

Overall, E-bikers without helmet suffered from any kind of intracranial bleeding in 60% of cases compared to 18.2% of patients with head protection (*p* = 0.0430). This rate is also significantly higher when compared to non-head-protected bicyclists, where intracranial bleedings occurred in 26.0% of cases (*p* = 0.0405). No difference was observed between head-protected accidents of E-bikers and bicyclists (E/H, 18.2%; B/H, 1.7%; *p* = 0.787). SDH was the most dominant (60%) and significantly most severe (AIS of 1.87) intracranial bleeding non-helmeted E-bikers suffered compared to helmeted (H) E-bikers (SDH, 9.1%, *p* = 0.006; AIS 0.27, *p* < 0.006) and non-helmeted (NH) bicyclist (SDH, 13.0%, *p* = 0.006; AIS, 0.4, *p* < 0.006). The occurrence of EDH and SAH did not differ significantly between helmeted and non-helmeted E-bikers after adjustment for multiple comparisons, even though the mean AIS for SAH in non-helmeted E-bikers was greater (*p* = 0.0208) than in non-helmeted bicyclists (AIS SAH: E = 0.93, B = 0.32). Also, a higher incidence of intracerebral hemorrhage of 46.7% with a mean AIS of 1.4 was present in the non-helmeted E-biker group compared to an incidence of 9.1% with a mean AIS of 0.27 in the helmeted group (ICH: *p* = 0.0430; AIS: *p* = 0.0107). Injury incidence and severity were higher in patients of the non-helmeted E-bike group compared to patients of the non-helmeted bicycle group (B/NH, ICH, 13.6%, *p* = 0.006; AIS, 0.41, *p* < 0.006). Cranial osseous injuries tended to be more severe in the non-helmeted E-bike group with a mean AIS of 1.2 than in the helmeted E-biker group (AIS, 0.36, *p* = 0.0813) and more severe than in non-helmeted bicyclists (AIS, 0.59, *p* = 0.129) but remained non-significant. Overall, craniocerebral injury occurrence and severity of the individual injury (AIS) were higher in the bicyclist group without helmet compared to helmeted bicyclists, which is explained in detail in the supplements (S4).

No significant differences in craniocerebral injury rate and bleeding severities could be found when comparing the non-helmeted and helmeted patients of the E-bike group with their equivalent of the motorcycle group (*p* > 0.05; S5-6). Motorcyclists without head protection had overall more frequent and more severe intracerebral injuries than patients wearing a helmet. More detailed information is presented in the supplements (S5-6).

**Fig. 4:**
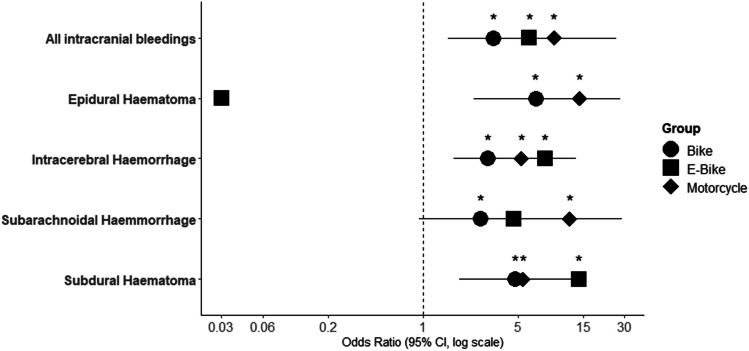
Odds ratio for unhelmeted vehicle users to suffer intracranial injures (adjusted for age). Asterisks indicate significantly increased risk of corresponding hemorrhage

Adjusted odds ratios for the occurrence of intracranial bleedings were calculated twofold. The odds ratios for all types of intracranial bleeding dependent on helmet use were adjusted for age (Table 2, Fig. 4). E-bikers without a helmet presented an OR of 6.0 (95% CI, 1.52 to 26.25, *p* = 0.00124) of suffering any kind of intracranial bleeding compared to bicyclists (OR, 3.29; 95% CI, 2.12 to 5.26, *p* < 0.00001) and motorcyclists (OR 9.13 95% CI, 3.63 to 25.36, *p* < 0.00001). Non-helmeted E-bikers presented an OR of 13.86 (95% CI, 2.96 to 85.55, *p* = 0.0017) for suffering an SDH (B = OR, 4.74; 95% CI, 2.41 to 10.46, *p* = 0.00003; M = OR, 5.40, 95% CI, 1.85 to 14.90, *p* = 0.0013). The OR for E-bikers without helmet protection was also significantly higher for SAH and ICH (Table 2). The odds ratios for epidural hematoma could not be calculated for the E-bike group due to little incidence. Age as a factor itself when adjusted to helmet use did not result in a significant finding in the E-bike group. Significant odds ratios for the parameter “Age” when adjusted for helmet use were solely found in the bicycle and motorcycle group but did not reach below an adjusted odds ratio of 0.96 in those cases, which might indicate a minimal clinical relevance despite statistical significance (Table 2).

**Table 2:** Adjusted odds ratios

Odds ratio (adjusted)		No helmet (adjusted for age)			Age (adjusted for helmet usage)		
Outcome		E-bike	Bike	Motorcycle	E-bike	Bike	Motorcycle
All bleedings	OR	6.00	3.29	9.13	0.97	0.97	0.99
	95%CI	1.52–26.25	2.12–5.26	3.63–25.36	0.92–1.01	0.96–0.98	0.97–1.00
	*p*	**0.0124**	** < 0.00001**	** < 0.00001**	0.13	** < 0.00001**	0.13
Subdural hematoma	OR	13.86	4.74	5.40	0.96	0.97	0.92
	95%CI	2.96–84.55	2.41–10.46	1.85–14.90	0.9–1.01	0.96–0.98	0.96–1.40
	*p*	**0.0017**	**0.00003**	**0.0013**	0.12	**0.000024**	0.09
Epidural hematoma	OR	0.0	6.68	14.02	1.0	1.0	1.0
	95%CI	NA-infinite	2.367–28.0	3.48–57.68	0.85–1.15	0.98–1.02	0.96–1.05
	*p*	1.00	**0.0018**	**0.00016**	0.99	0.689	0.99
Subarachnoid hemorrhage	OR	4.65	2.64	11.88	1.0	0.96	0.96
	95%CI	0.94–28.84	1.54–4.75	4.36–33.70	0.845–1.15	0.95–0.98	0.93–0.98
	*p*	0.065	**0.0007**	** < 0.00001**	0.99	** < 0.00001**	**0.0004**
Intracerebral hemorrhage	OR	7.83	2.97	5.23	0.98	0.98	1.01
	95%CI	1.71–44.12	1.67–5.66	1.98–13.33	0.93–1.03	0.97–0.99	0.98–1.03
	*p*	**0.011**	**0.0004**	**0.0006**	0.38	**0.0015**	0.61

### Matched pair subgroup analysis

A subgroup analysis via propensity score matching for age, sex, and helmet use in between the bike and E-bike group in a 4:1 ratio was performed to confirm the previous findings respective the baseline differences. Matching was successful (S7-8) with no significant differences in between the relevant baseline characteristics. There was also no significant difference in between occurrence of bleedings overall, epidural hematoma, or subarachnoid bleedings. Yet, this analysis confirms the prior findings of a significantly increased occurrence of intracerebral bleedings (B = 8.9% vs. E = 20.8%, *p* = 0.037) and subdural hematoma in the E-bike compared to the bicycle group (B = 7.6% vs. E = 25.0%, *p* = 0.002) (Table 3).

**Table 3:** Matched pair subgroup analysis E-bike vs. bike (1:4 ratio) (matching according to age, helmet usage, and sex)

Propensity scored matching	Bike (B)	E-bike (E)	*p*-value (not adjusted)
*N*	192	48	
Sex (male) (*n*, %)	111 (77.1%)	31 (64.6%)	0.211
Age (mean/SD)	54.72 (± 15.75)	54.96 (± 16.34)	0.929
BMI (mean/SD)	24.22 (± 3.92)	24.83 (± 3.95)	0.348
Helmet (yes) (*n*, %)	138 (71.9%)	33 (68.8%)	0.722
All intracranial bleedings (yes) (*n*, %)	41 (21.4)	15 (31.2)	0.181
Epidural hematoma (yes) (*n*, %)	7 (3.6)	1 (2.1)	1
Intracerebral bleeding (yes) (*n*, %)	17 (8.9)	10 (20.8)	0.037
Subarachnoid bleeding (yes) (*n*, %)	131 (16.1)	8 (16.7)	1
Subdural hematoma (yes) (*n*, %)	15 (7.8)	12 (25.0)	0.002

### Cervical spine injuries

Different accident and injury patterns to the cervical spine were compared in between the vehicle groups. After adjustment for multiple testing, no significance was reached in terms of accident mechanisms as hyperextension/flexion, distraction, distortion, and contusion; there was no difference in between the groups except injuries to the processus transversi when comparing E-bikers and motorcyclists (E = 1.1% vs. M = 0%; *p* = 0.006). In addition, incidence and severity of spinal cord and osseous injuries on the cervical level did not show significant differences to the bicyclist and motorcycle group (S9).

### Craniomaxillofacial injuries

Injury severity in terms of AIS and osseous injuries to the midface were analyzed. Injuries to the facial soft tissue were significantly elevated in the E-biker group compared to motorcyclists (AIS: E = 0.35 vs. M = 0.16, *p* = 0.011). Further information can be found in the supplements (S0).

### Outcome

Outcome parameters defined as ICU admission, rehabilitation, and mortality have been analyzed. Motorcyclists had a significantly higher rate of ICU admission (45.7%) compared to E-bikers (27.1%) which remained not significant after adjustment for multiple testing. Performed rehabilitation showed significant differences (M = 36.4% vs. E = 16.7%, *p* = 0.0495). Mortality was comparable between all groups (B = 1.9%, E = 2.1%, M = 3.6%) (Table 4).

**Table 4:** Outcomes

Outcome	Bike	E-bike	Motorcycle	*p*-value (adj.) (E vs. B)	*p*-value (adj.) (E vs. M)
ICU (*n*, %)	131 (17.7%)	13 (27.1%)	128 (45.7%)	0.393	0.0819
Rehabilitation (*n*, %)	78 (10.5%)	8 (16.7%)	102 (36.4%)	0.6270	**0.0495**
Death (*n*, %)	14 (1.9%)	1 (2.1%)	10 (3.6%)	1.0	1.0

## Discussion

Head injuries account for up to 70% of potentially fatal injuries caused by bicycles [14]. Thereby, the use of preventive measures and protection is important to consider. Historically, it was thought that bicycle riders that are protected by helmets have a low risk of severe head trauma, a situation that may have changed along with the development of electric drives with powerful engines [10, 15]. In this respect, our study has yielded the following main results:

1. The overall incidence and severity of cranio-cervical and traumatic brain injuries resulting from E-bike accidents are similar to that of motorcycle accidents.
2. The odds for E-bikers not wearing helmets to suffer an intracranial hemorrhage in traffic accident is increased sixfold, with a 13.86-fold increase for the odds of sustaining a subdural hemorrhage.
3. Older age alone did not result in a significantly increased risk of intracranial hemorrhage in the E-bike group, but in certain types of hemorrhage in the other vehicle groups. However, clinical/practical relevance might be minimal.

We feel that our results are in keeping with the current literature: Although bicycle helmet use has been shown to reduce the odds of head injury, including serious and fatal head injury [16, 17], helmet use remains considerably low among bike and E-bike users sustaining injuries [18, 19]. This is also reflected in our data, which show a rate of 33.8% helmet use in conventional bicyclists admitted to our hospital with cranio-cervical and traumatic brain injuries in the years of 2009–2019. Helmet use rates in our cohort of patients admitted after accidents with E-bikes (68.8%) and motorcycles (91.8%) are higher, but still need improvement. This is especially underlined by our results suggesting a sixfold increase of the odds of non-helmeted E-bikers to suffer from intracranial hemorrhage in case of an accident. Furthermore, our data depict a 13.86-fold increase in odds for sustaining a subdural hemorrhage in case of an E-bike crash without helmet use. These increased risks have been confirmed in an additional matched pair subgroup analysis in our manuscript. This might be reflected in the lower median GCS at hospital admission after E-bike accident (GCS = 13) compared to conventional bicycle accident (GCS = 14) shown by our data, which did not remain significant after adjustment for multiple comparisons. However, median GCS at admission was comparable to patients after motorcycle accident (GCS = 13), which might suggest comparable injury severity between E-bike and motorcycle accidents. A further influential factor suggested by our data is patient age. Patients admitted after E-bike accident were significantly older than patients who sustained a bicycle or motorcycle accident. Generally, E-bikes are highly attractive for the elderly, as motor support promotes mobility not provided by conventional bicycles and are believed to be safer due to lower speeds than motorcycles with easier accessibility. However, E-bike riders of 55 years of age and older have been shown to be associated with the highest likelihood of severe injury [20]. Additionally, among other age-associated risk factors, a high proportion of the older population is under anticoagulant medication for various reasons, which is associated with an increased risk of cerebral hemorrhage even without an accident [21]. Due to the fact that anticoagulant usage could not be extracted from our data, the odds for sustaining an intracranial bleeding due to increasing age were adjusted by helmet usage. This analysis did not yield E-bike-specific significances, yet the actual number of patients with regular intake of anticoagulants is still unknown. Still, we propose to consider this patient cohort as especially endangered. Thus, while adequate protective gear is generally recommended when operating two-wheelers in traffic, E-bike users should be strongly advised to wear proper helmets due to their demographics, increased accident risk, and high risk of sustaining an intracranial, especially subdural, hemorrhage when having an accident.

We found a significantly higher incidence of in soft tissue injuries to the face. The most likely explanation for this finding is helmet worn and type of helmet. In literature, the protective effect of standard bicycle helmets against midfacial injuries is discussed controversially [22, 23]. Full-face helmets, on the other hand, offer full-face protection, true to their name [24]. They are widely used by motorcyclists, but with exception of competitive mountain biking, its use among E-bikers is rare.

The highest mean age of the E-biker group could further contribute to the increase in soft tissue injuries in the midface area. With age, the defense and catching reflexes slow down compared to younger age, with consecutive increase in midfacial trauma [25, 26]. In addition, the age group above 60 years wears a helmet less often compared to the Swiss average (50% vs. 56%) [27].

Due to the overall small number of midface injuries in our cohort and small E-biker group compared to cycling group, a final conclusive analysis is not possible. By analyzing larger amounts of data, an accumulation in the elderly and therefore in the E-biker group could be expected.

In our study, the only significant difference in injury pattern of the cervical spine was seen in fractures of the articular process between the E-bike and motorcycle group, whereas the overall injury presence was low in all groups. A recent publication by Wu et al. describes the spinal injury pattern in E-bikes and report that fractures of the spine occurred less often in the cervical spine as in the thoracolumbar region [28]. In their population, injuries to the lower cervical spine also occurred more often than in the upper section, which is also displayed in our study findings. These spinal injury patterns are also reported in patients suffering motorcycle accidents [29]. This might be due to the fact that the riding posture is relatable in all three-vehicle groups. Another study analyzed the effect of helmet usage in traditional bicycle injuries on the cervical spine where no protective effect on the spine was seen [30]. Still, another study focusing on the same topic in a motorcycle population reports a decreased risk for cervical spine injuries for helmeted riders [31]. Therefore, it might be assumed that wearing a helmet should at least not have a negative impact on injuries to the cervical spine since it does not relevantly alter the posture of the neck, which results in a comparable injury mechanism.

### Strengths and limitations

The main limitation of this study is the low number of accidents involving E-bikes is not as high as for other modes, increases the risk of a type 2 error, and might therefore be underpowered especially in the occurrence of overall rare events. Another limitation of this study is the retrospective nature of the data collection, with all the associated limitations. Being a retrospective study, it is possible that the number of E-bikers has been underestimated.

Our groups presented differences in the baseline characteristics since different population groups tend to use either one of those. Statistical approaches have been used to account for it but certainly cannot totally resolve the underlying (not quantifiable) differences in those patient cohorts.

In addition, the attempt to divide all two-wheelers on the road into only three groups is a very pragmatic approach. Unfortunately, a more specific analysis of the intention of using the different vehicles could not be performed in this study. Meant hereby is that bicycles can be used for everyday mobility or more athletic activities (i.e., mountain biking, bike racing) that might be associated with a higher velocity and a difference in helmet usage. E-bikes are assumed to be used more for general mobility or outdoor activities but rarely for prior mentioned athletic events. These circumstances in usage have to be taken into account if transferring our results to the individual patient.

One of the strengths of this study is that it was conducted in a level I trauma center, where the severity of injuries varies widely. Victims from rural and urban areas were included. Information for this study was obtained directly from a detailed patient database. In contrast, other published studies have often relied solely on field reports from paramedics, insurance reports, or questionnaire-based surveys.

Statistical analysis was performed conservatively using the false discovery rate (FDR) for multiple comparisons. In this way, we adjusted for possible false positives according to the state of the art and assume that the results can be considered reliable given the comparatively small E-bike population. By performing the analysis of the odds of suffering an intracranial hemorrhage adjusted for the main factors that differ between our groups (age and helmet), we reduced the overall bias of our results.

Another strength of the present study is that the results were interpreted and discussed by a multidisciplinary group of researchers consisting of experienced specialists in trauma surgery, neurosurgery, cranio-maxillofacial and oral surgery, and neuroradiology. This emphasizes the clinical relevance of the results and conclusions.

## Conclusion

In summary, our study demonstrates that the overall pattern of head and cervical injuries in E-bikers is very similar to that of those induced by high-energy motorcycle injuries. As they are used by a more senior population, multiple risk factors (i.e., anticoagulation) apply in terms of complications and secondary intracranial bleeding. We feel that preventive measures should be reinforced, i.e., use of helmets to prevent from intracranial injury.

## Supplementary Information

Below is the link to the electronic supplementary material.

Supplementary file1 (DOCX 43 KB)

## Data Availability

No datasets were generated or analyzed during the current study.

## Abbreviations

AIS: Abbreviated Injury Scale
BMI: Body mass index
EDH: Epidural hematoma
DAI: Diffuse axonal injury
FDR: False discovery rate
GCS: Glasgow Coma Scale
ICD: International Classification of Diseases
ICH: Intracerebral hemorrhage
ICU: Intensive care unit
SAH: Subarachnoid hemorrhage
SDH: Subdural hematoma
TBI: Traumatic brain injury
