# Supplementary material for: Cranio-cervical and traumatic brain injury patterns—do they differ between electric bicycle, bicycle, and motorcycle-induced accidents?
Source: Eur J Trauma Emerg Surg. 2024 Apr 9;50(6):3039–48. doi: 10.1007/s00068-024-02510-1 (PMC11666674; doi:10.1007/s00068-024-02510-1)
Supplement: Supplementary file 1 — Supplementary file1 (DOCX 43 KB) [file 68_2024_2510_MOESM1_ESM.docx]

**Supplementary Material**

|  | **Bike (B)** | **E-Bike (E)** | **Motorcycle (M)** | **p-value (adj.) (E vs. B)** | **p-value (adj.) (E vs. M)** |
| --- | --- | --- | --- | --- | --- |
| **Season** | | | | 1.0 | 0.77 |
| Spring (March-Mai) (n,%) | 215 (29.1%) | 11 (22.9%) | 87 (31.1%) |  | |
| Summer (June-August) (n,%) | 293 (39.6%) | 21 (43.8%) | 111 (39.6%) |  |  |
| Fall (September-November) (n,%) | 173 (23.4%) | 12 (25.0%) | 71 (25.4%) |  |  |
| Winter (December-February (n,%) | 59 (8.0%) | 4 (8.3%) | 11 (3.9%) |  |  |
| **Light conditions** | | | | 1.0 | 1.0 |
| Dawn (n,%) | 39 (5.3%) | 3 (6.2%) | 11 (4.0%) |  | |
| Daylight (n,%) | 520 (70.8%) | 36 (75.0%) | 215 (77.6%) |  |  |
| Darkness (n,%) | 175 (23.8%) | 9 (18.8%) | 51 (18.4%) |  |  |
| External influence (n,%) | 209 (28.2%) | 8 (16.7%) | 141 (50.4%) | 0.33 | **<0.006** |

*S1: Injury circumstances*

|  | **Bike** | **E-Bike** | **Motorcycle** | **p-value (adj.) (E vs. B)** | **p-value (adj.) (E vs. M)** |
| --- | --- | --- | --- | --- | --- |
| **TBI** | | | | 0.419 | 0.849 |
| Mild (n,%) | 634 (85.7%) | 37 (77.1%) | 205 (73.2%) |  | |
| Moderate (n,%) | 45 (6.1%) | 7 (14.6%) | 28 (10.0%) |  |  |
| Severe (n,%) | 34 (4.6%) | 2 (4.2%) | 31 (11.1%) |  |  |
| Open (n,%) | 9 (1.2%) | 0 (0.0%) | 3 (1.1%) |  |  |
| None (n,%) | 18 (2.4%) | 2 (4.2%) | 13 (4.6%) |  |  |
| GCS (Mean/SD) | 14.05 (±2.27) | 13.27 (±2.88) | 13.16 (±3.41) | 0.0819 | 1.0 |
| Bleeding (all) | 157 (21.2%) | 15 (31.2%) | 73 (26.1%) | 0.391 | 0.895 |
| **Subdural Haematoma** | | | | | |
| Yes (n,%) | 73 (9.9%) | 12 (25.0%) | 30 (10.7%) | **0.0107** | **0.0495** |
| AIS (Mean/SD) | 0.30 (±0.93) | 0.77 (±1.36) | 0.34 (±1.00) | **0.006** | **0.0405** |
| **Epidural Haematoma** | | | | | |
| Yes (n,%) | 40 (5.4%) | 1 (2.1%) | 10 (3.6%) | 0.849 | 1.0 |
| AIS (Mean/SD) | 0.17 (±0.72) | 0.06 (±0.43) | 0.11 (±0.61) | 0.649 | 0.895 |
| **Subarachnoid haemorrhage** | | | | | |
| Yes (n,%) | 88 (11.9%) | 8 (16.7%) | 40 (14.3%) | 0.806 | 1.0 |
| AIS (Mean/SD) | 0.26 (±0.75) | 0.42 (±1.01) | 0.36 (±0.92) | 0.432 | 0.994 |
| **Intracerebral haemorrhage** | | | | | |
| Yes (n,%) | 80 (10.8%) | 10 (20.8%) | 36 (12.9%) | 0.1890 | 0.502 |
| AIS (Mean/SD) | 0.26 (±0.74) | 0.42 (±1.01) | 0.36 (±0.92) | 0.129 | 0.399 |
| Bone Injury AIS (Mean/SD) | 0.45 (±1.00) | 0.62 (±1.20) | 0.36 (±0.94) | 0.561 | 0.259 |
| **Edema** | | | | | |
| Yes (n,%) | 13 (1.8%) | 0.00 (0%) | 6 (2.1%) | 1.0 | 0.986 |
| AIS (Mean/SD) | 0.06 (±0.48) | 0.00 (±0) | 0.06 (±0.44) | 0.732 | 0.649 |
| **Diffuse axonal injury** | | | | | |
| Yes (n,%) | 2 (0.3) | 0.00 (0%) | 4 (1.4%) | 1.0 | 1.0 |
| AIS (Mean/SD) | 0.01 (±0.21) | 0.00 (±0) | 0.06 (±0.48) | 1.0 | 0.75 |
| **Intracerebral sinus injury** | | | | | |
| Yes (n,%) | 0 (0.00% | 0.00 (0%) | 0.00 (0%) | 1.0 | N/A |
| AIS (Mean/SD) | 0.01 (±0.24) | 0.00 (±0) | 0.00 (±0) | 0.986 | N/A |
| Pneumocephalus (n, %) | 40 (5.4%) | 1 (2.1%) | 8 (2.9%) | 0.849 | 1.0 |
| Artery injury (n, %) | 4 (0.05%) | 1 (2.1%) | 3 (1.1%) | 1.0 | 1.0 |
| A. Vertebralis, AIS (Mean/SD) | 0.01 (±0.19) | 0.06 (±0.43) | 0.01 (±0.18) | 0.324 | 0.399 |
| A. Carotis interna, AIS (Mean/SD) | 0.00 (±0.11) | 0.00 (±0) | 0.02 (±0.25) | 1.0 | 0.891 |

*S2: Cranial injuries*

|  | **E-Bike** | | | **Bike** | | | **E vs. B** | **E vs. B** |
| --- | --- | --- | --- | --- | --- | --- | --- | --- |
|  | **No Helmet** | **Helmet** | **p-value (E) (adj.)** | **No Helmet** | **Helmet** | **p-value (B) (adj.)** | **p-value (adj.)**  **(No Helmet)** | **p-value (adj.) (Helmet)** |
| Sex (Male) | 9 (60%) | 22 (66.7%) | 1.00 | 318 (65.6%) | 188 (75.8%) | **0.0305** | 1.0 | 0.721 |
| Age (Mean/SD) | 59.60 (±17.96) | 52.85 (±15.37) | 0.446 | 40.73 (±16.56) | 45.89 (±16.4) | **<0.006** | **<0.006** | 0.0807 |
| **TBI, n (%)** |  | | **0.0338** |  | | **<0.006** | **0.006** | 1.0 |
| Mild | 8 (53.3%) | 29 (87.9%) |  | 419 (86.4%) | 209 (84.3%) |  | | |
| Moderate | 5 (33.3%) | 2 (6.1%) |  | 31 (6.4%) | 14 (5.6%) |  |  |  |
| Severe | 2 (13.3%) | 0 (0.0%) |  | 25 (5.2%) | 8 (3.2%) |  |  |  |
| Open | 0 (0%) | 0 (0%) |  | 7 (1.4%) | 2 (0.8%) |  |  |  |
| None | 0 (0.0) | 2 (6.1%) |  | 3 (0.6%) | 15 (6.0%) |  |  |  |
| GCS (Mean/SD) | 11.27 (±4.27) | 14.18 (±1.21) | **0.006** | 14.00 (±2.31) | 14.19 (±2.12) | 0.6140 | **<0.006** | 1.0 |

*S3: Helmet – TBI – E-bike/Bike*

|  | **E-Bike (E)** | | | **Bike (B)** | | | **E vs. B** | **E vs. B** |
| --- | --- | --- | --- | --- | --- | --- | --- | --- |
|  | **No Helmet** | **Helmet** | **p-value (E) (adj.)** | **No Helmet** | **Helmet** | **p-value (B) (adj.)** | **p-value (adj.)**  **(No Helmet)** | **p-value (adj.) (Helmet)** |
| **Bleeding (all)** | 9 (60%) | 6 (18.2%) | **0.0430** | 126 (26.0%) | 29 (11.7%) | **<0.006** | **0.0405** | 0.787 |
| **Subdural Haematoma** | | | | | | | | |
| Yes (n,%) | 9 (60%) | 3 (9.1%) | **0.006** | 63 (13.0%) | 9 (3.6%) | **<0.006** | **0.006** | 0.661 |
| AIS (Mean/SD) | 1.87 (±1.60) | 0.27 (±0.88) | **<0.006** | 0.40 (±1.04) | 0.11 (±0.56) | **<0.006** | **<0.006** | 0.391 |
| **Epidural Haematoma** | | | | | | | | |
| Yes (n,%) | 0 (0.0%) | 1 (3.0%) | 1.00 | 36 (7.4%) | 3 (1.2%) | **0.006** | 0.891 | 1.0 |
| AIS (Mean/SD) | 0 (±0.0) | 0.09 (±0.52) | 0.849 | 0.24 (±0.84) | 0.04 (±0.33) | **<0.006** | 0.627 | 0.75 |
| **Subarachnoid haemorrhage** | | | | | | | | |
| Yes (n,%) | 5 (33.3%) | 3 (9.1%) | 0.281 | 69 (14.2%) | 18 (7.3%) | **0.0377** | 0.276 | 1.0 |
| AIS (Mean/SD) | 0.93 (±1.49) | 0.18 (±0.58) | 0.0594 | 0.32 (±0.80) | 0.16 (±0.59) | **0.0305** | **0.0208** | 1.0 |
| **Intracerebral haemorrhage** | | | | | | | | |
| Yes (n,%) | 7 (46.7%) | 3 (9.1%) | **0.0430** | 66 (13.6%) | 14 (5.6%) | **0.0107** | **0.006** | 1.0 |
| AIS (Mean/SD) | 1.40 (±1.55) | 0.27 (±0.88) | **0.0107** | 0.41 (±1.05) | 0.17 (±0.69) | **0.006** | **<0.006** | 0.787 |
| **Bone injury AIS (Mean/SD)** | 1.20 (±1.42) | 0.36 (±0.99) | 0.0813 | 0.59 (±1.11) | 0.16 (±0.62) | **<0.006** | 0.129 | 0.303 |

*S4: E-bike/bike bleedings*

|  | **E-Bike (E)** | | | **Motorcycle (M)** | | | **E vs, M** | **E vs. M** |
| --- | --- | --- | --- | --- | --- | --- | --- | --- |
|  | No Helmet | Helmet | p-value (E) (adj.) | No Helmet | Helmet | p-value (M) (adj.) | p-value (adj.) (No Helmet) | p-value (adj.) (Helmet) |
| Sex (Male) | 9 (60%) | 22 (66.7%) | 1.0 | 21 (91.3%) | 229 (89.1%) | 1.000 | 0.182 | **0.006** |
| Age (Mean/SD) | 59.60 (±17.96) | 52.85 (±15.37) | 0.446 | 32.87 (±15.07 | 40.84 (±16.06) | 0.0813 | **<0.006** | **<0.006** |
| **TBI** |  | | **0.0338** |  | | **<0.006** | 0.71 | 0.743 |
| Mild | 8 (53.3%) | 29 (87.9%) |  | 9 (39.1%) | 196 (76.3%) |  | | |
| Moderate | 5 (33.3%) | 2 (6.1%) |  | 5 (21.7%) | 23 (8.9%) |  |  |  |
| Severe | 2 (13.3%) | 0 (0.0%) |  | 7 (30.4%) | 24 (9.3%) |  |  |  |
| Open | 0 (0%) | 0 (0%) |  | 2 (8.7%) | 1 (0.4%) |  |  |  |
| None | 0 (0.0) | 2 (6.1%) |  | 0 (0.0%) | 13 (5.1%) |  |  |  |
| GCS (Mean/SD) | 11.27 (±4.27) | 14.18 (±1.21) | **0.006** | 9.87 (±4.98) | 13.45 (±3.08) | **<0.006** | 0.7328 | 0.432 |

*S5: Helmet – TBI – E-Bike/Motorcycle*

|  | **E-Bike (E)** | | | **Motorcycle (M)** | | | **E vs. M** | **E vs. M** |  |
| --- | --- | --- | --- | --- | --- | --- | --- | --- | --- |
|  | **No Helmet** | **Helmet** | **p-value (E) (adj.)** | **No Helmet** | **Helmet** | **p-value (M) (adj.)** | **p-vale (adj.) (No Helmet)** | **p-value (adj.) (Helmet)** |  |
| **Bleeding (all)** | 9 (60%) | 6 (18.2%) | **0.0430** | 16 (69.6%) | 57 (22.2%) | **<0.006** | 1.0 | 1.0 |  |
| **Subdural Haematoma** | | | | | | | | | |
| Yes (n,%) | 9 (60%) | 3 (9.1%) | **0.006** | 7 (30.4%) | 23 (8.9%) | **0.025** | 0.39 | 1.0 |  |
| AIS Mean/SD) | 1.87 (±1.60) | 0.27 (±0.88) | **<0.006** | 1.04 (±1.66) | 0.28 (±0.90) | **<0.006** | 0.388 | 1.0 |  |
| **Epidural Haematoma** | | | | | | | | | |
| Yes (n,%) | 0 (0.0%) | 1 (3.0%) | 1.00 | 5 (21.7%) | 5 (1.9%) | **<0.006** | 0.391 | 1.0 |  |
| AIS (Mean/SD) | 0 (±0.0) | 0.09 (±0.52) | 0.849 | 0.74 (±1.48) | 0.06 (±0.42) | **<0.006** | 0.195 | 0.994 |  |
| **Subarachnoid haemorrhage** | | | | | | | | | |
| Yes (n,%) | 5 (33.3%) | 3 (9.1%) | 0.281 | 11 (47.8%) | 29 (11.3%) | **<0.006** | 0.895 | 1.0 |  |
| AIS (Mean/SD) | 0.93 (±1.49) | 0.18 (±0.58) | **0.0594** | 1.22 (±1.38) | 0.28 (±0.83) | **<0.006** | 0.891 | 0.8490 |  |
| **Intracerebral haemorrhage** | | | | | | | | | |
| Yes (n,%) | 7 (46.7%) | 3 (9.1%) | **0.0430** | 9 (39.1%) | 27 (10.5%) | **<0.006** | 0.901 | 1.0 |  |
| AIS (Mean/SD) | 1.40 (±1.55) | 0.27 (±0.88) | **0.0107** | 1.22 (±1.57) | 0.32 (±0.93) | **<0.006** | 0.726 | 1.0 |  |
| **Bone injury AIS (Mean/SD)** | 1.20 (±1.42) | 0.36 (±0.99) | **0.0813** | 1.30 (±1.49) | 0.28 (±0.82) | **<0.006** | 0.831 | 0.895 |  |

*S6: Helmet Motorcycle E-bike bleeding*


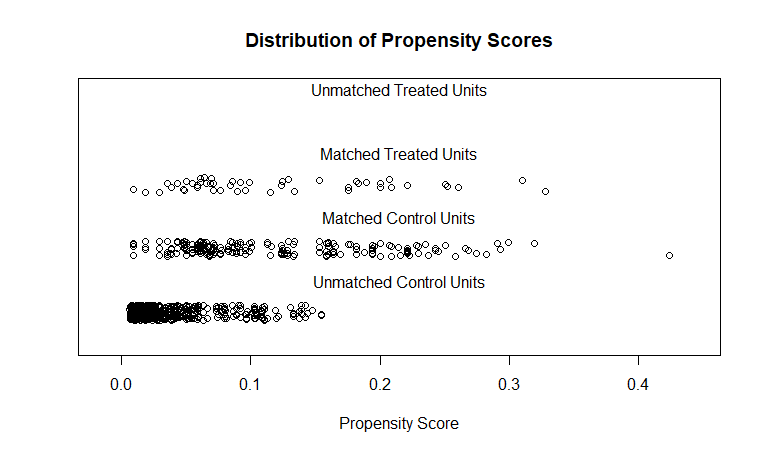


S7: Propensity score matching


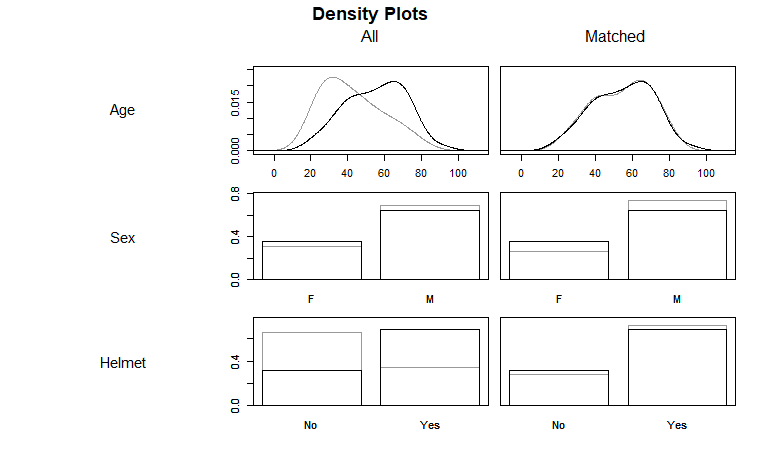


S8: Matching density plots

|  | **Bike (B)** | **E-Bike (E)** | **Motorcycle (M)** | **p-value (adj.)**  **E vs. B** | **p-value (adj.)**  **E vs. M** |
| --- | --- | --- | --- | --- | --- |
| Cervical Spine (n,%)* | 94 (12.7%) | 9 (18.8%) | 50 (17.9%) | 0.67 | 1.0 |
| Upper Cervical Spine (n,%) | 17 (2.3%) | 4 (8.3%) | 10 (3.6%) | 0.130 | 0.603 |
| Lower Cervial Spine (n,%) | 61 (8.2%) | 5 (10.4%) | 29 (10.4%) | 1.0 | 1.0 |
| Hyperextension/Flexion (n,%) | 16 (2.2%) | 1 (2.1%) | 7 (2.5%) | 1.0 | 1.0 |
| Distraction (n,%) | 8 (1.1%) | 0.00 (0%) | 3 (1.1%) | 1.0 | 1.0 |
| Distorsion (n,%) | 14 (1.9%) | 2 (4.2%) | 12 (4.3%) | 0.895 | 1.0 |
| Contusion (n,%) | 9 (1.2%) | 0.00 (0%) | 5 (1.8%) | 1.0 | 1.0 |
| Bone/ligament AIS (Mean/SD) | 0.05 (±0.34) | 0.12 (±0.49) | 0.06 (±0.35) | 0.423 | 0.649 |
| **Spinal cord Injury** | | | | | |
| Yes (n,%) | 13 (1.8%) | 1 (2.1%) | 5 (1.8%) | 1.0 | 1.0 |
| AIS (Mean/SD) | 0.06 (±0.49) | 0.06 (±0.43) | 0.08 (±0.61) | 1.0 | 1.0 |
| Ligaments (n,%) | 11 (1.5%) | 1 (2.1%) | 1 (0.4%) | 1.0 | 0.994 |
| **Osseus injuries** | | | | | |
| Atlas (n,%) | 8 (1.1%) | 1 (2.1%) | 4 (1.4%) | 1.0 | 1.0 |
| Axis (n,%) | 9 (1.2%) | 2 (4.2%) | 6 (2.1%) | 0.635 | 1.0 |
| Corpus Axis (n,%) | 9 (1.2%) | 2 (4.2%) | 5 (1.8%) | 0.635 | 0.925 |
| Dens Axis (n,%) | 7 (0.9%) | 1 (2.1%) | 4 (1.4%) | 1.0 | 1.0 |
| Proc. Articularis (n,%) | 8 (1.1%) | 3 (6.2%) | 0 (0.0%) | 0.07620 | **0.006** |
| Proc. Transversus (n, %) | 6 (0.8%) | 1 (2.1%) | 7 (2.5%) | 1.0 | 1.0 |
| Proc. Spinosus (n,%) | 25 (3.4%) | 0 (0%) | 10 (3.6%) | 0.733 | 0.732 |
| Discus intervertebralis (n,%) | 12 (1.6%) | 2 (4.2%) | 0 (0%) | 0.816 | 0.0594 |

*S9: Cervical spine injuries*

|  | **Bike** | **E-Bike** | **Motorcycle** | **p-value (adj.)**  **(E vs. B)** | **p-value (adj.)**  **(E vs. M)** |
| --- | --- | --- | --- | --- | --- |
| Bone face AIS (Mean/SD) | 0.45 (±1.00) | 0.58 (1.07±) | 0.48 (±1.04) | 0.7327 | 0.861 |
| Le Fort (n,%) | 11 (1.5%) | 1 (2.1%) | 7 (2.5%) | 1.0 | 1.0 |
| Mouth AIS (Mean/SD) | 0.12 (±0.34) | 0.10 (±0.31) | 0.07 (±0.27) | 0.994 | 0.743 |
| Nose AIS (Mean/SD) | 0.12 (±0.37) | 0.21 (±0.54) | 0.08 (±0.31) | 0.385 | 0.0738 |
| Ear AIS (Mean/SD) | 0.02 (±0.15) | 0.00 (±0) | 0.01 (±0.08) | 0.7320 | 0.891 |
| Eye (n,%) | 12 (1.6%) | 0.00 (0%) | 1 (0.4%) | 1.0 | 1.0 |
| Soft tissue Face AIS (Mean/SD) | 0.41 (±0.50) | 0.35 (±0.53) | 0.16 (±0.37) | 0.8160 | 0.011 |

*S10: Cranio-maxillo-facial injuries*
